# Trends and determinants of anemia in children 6–59 months and women of reproductive age in Chad from 2016 to 2021

**DOI:** 10.1186/s40795-023-00777-y

**Published:** 2023-10-23

**Authors:** Eleonor Zavala, Sarah Adler, Edgar Wabyona, Martin Ahimbisibwe, Shannon Doocy

**Affiliations:** 1 Johns Hopkins Bloomberg School of Public Health, 615 N Wolfe St, Baltimore, MD 21205 USA; 2 World Food Programme Chad, N’Djamena, Chad

**Keywords:** Chad, Anemia, Micronutrient deficiency, Child nutrition, Maternal nutrition

## Abstract

**Background:**

Hemoglobin assessments in children and women have been conducted annually in Chad since 2016 through the Standardized Monitoring and Assessment of Relief and Transitions (SMART) cross-sectional surveys. This analysis aims to characterize national and sub-national trends in anemia among children under five and women of reproductive age from 2016 to 2021 and to compare risk factors for anemia before and during the COVID-19 pandemic.

**Methods:**

Hemoglobin concentrations were measured in approximately half of the 12,000 to 15,000 included households each year, except for 2020 when hemoglobin tests were omitted. For children 6 to 59 months of age, anemia was defined as hemoglobin less than 11.0 g/dL. Anemia was defined as hemoglobin less than 11.0 g/dL and 12.0 g/dL for pregnant women and non-pregnant women, respectively. Trends were stratified by agroecological zone, and tests of proportions were used to assess statistical significance. Simple and multivariate logistic regression models were conducted for 2019 and 2021 to identify risk factors for anemia.

**Results:**

Reductions in anemia over the 6-year period were significant among women (47.6–30.8%, p = 0.000) and children (68.6–59.6%, p = 0.000). The Sudanian zone had consistently higher rates, particularly in children, compared to the Sahelian and Saharan zones. Significant declines in women’s anemia were observed in all zones from 2019 to 2021, but this global decline was not observed among children, where rates in the Saharan zone significantly increased. In 2019, only minimum dietary diversity significantly reduced the odds of anemia in children (AOR: 0.65, 95%CI: 0.46–0.92), whereas in 2021, improvements in all diet indicators were associated with lower odds of anemia. Improved household socio-economic factors, including head of household literacy, were associated with lower odds of anemia in children (2019 AOR: 0.76, 95%CI: 0.67, 0.88) and women (2019 AOR: 0.75, 95%CI: 0.65, 0.87; 2021 AOR: 0.81, 95%CI: 0.70, 0.93).

**Conclusions:**

Anemia declined significantly in Chad among women of reproductive age and children from 2016 to 2021, but the national prevalence of 60% among children remains unacceptably high. Sub-national differences in anemia rates underline the need to identify and address regional causes of anemia while strengthening national level programs.

**Supplementary Information:**

The online version contains supplementary material available at 10.1186/s40795-023-00777-y.

## Background

Anemia affects 269 million children and 570 million women of reproductive age globally, with disproportionately higher rates in South Asia and Sub-Saharan Africa [1]. Anemia contributes to poor cognitive and motor development and is linked with lower school attainment and behavioral problems later in life [2, 3]. In pregnancy, anemia is associated with adverse birth and obstetric outcomes, including preterm birth, low birth weight and postpartum hemorrhage [4]. Iron deficiency is regarded as a major cause of anemia, but other causes, including infectious diseases, hemoglobinopathies, and other nutritional deficiencies may contribute significantly across different populations and settings [5].

**Fig. 1:**
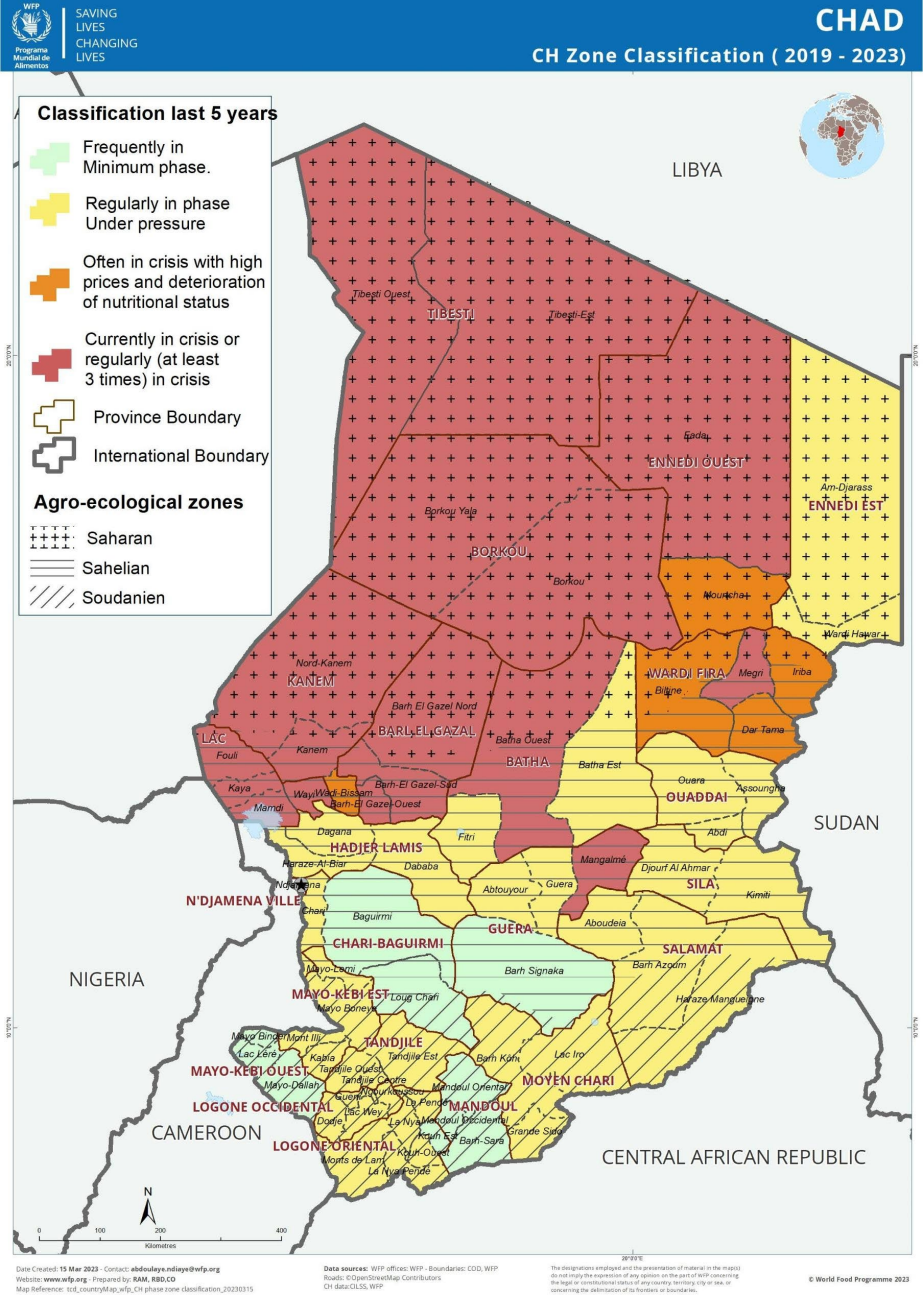
Chad agroecological zones map

Chad is a landlocked country in Central Africa that is characterized by three climatic zones: the Saharan desert to the north, the Sahel in the middle, and the Sudanian savanna in the south (Fig. 1). The economy relies heavily on agro-pastoral activities but suffers from frequent climatic hazards, political conflict, and intercommunal violence [6]. Poverty and food insecurity levels are high and were exacerbated during the COVID-19 pandemic [7]. In 2021, 10.9% of Chadian children under five were wasted and 30.4% were stunted, placing Chad among the high and very high prevalence thresholds set by World Health Organization (WHO) for wasting and stunting, respectively [8, 9]. Diets lacking in sufficiency and diversity contribute to micronutrient deficiencies. In Chad, 66% of children 6–59 months of age, and 45% of women of reproductive age were estimated to have anemia in 2019 [10, 11].

Evidence on the attributable fraction of the various causes of anemia in women and children in Chad are lacking. However, inadequate micronutrient intakes, including insufficient iron-rich diets, are likely contributors to the prevalence of anemia given the overall poor population nutritional status. Malaria is another major contributor to anemia and child mortality in sub-Saharan Africa [12], and is endemic in Chad’s Sahelian and Sudanian zones, with an estimated 3.19 million cases in 2019 [13]. In Chad, allele frequencies for sickle cell disease, a hemoglobinopathy that causes anemia, has been estimated to be present in 5% of neonates annually [14]. Additionally, helminth infections, including hookworm and *Ascaris lumbricoides*, have been associated with anemia in Chadian children and mothers [15]. More research is needed to understand the anemia landscape in Chad, including the contribution of other infectious diseases and micronutrient deficiencies such as folate and B12. This analysis describes national and sub-national trends in anemia in children 6–59 months of age and in women of reproductive age for the five-year period before and during the COVID-19 pandemic with the use of annual SMART surveys. We further explored determinants for anemia in both children and women in 2019 and 2021 to inform strategy and targeting for national multi-sectoral nutrition programming in the post-pandemic era.

## Methods

SMART surveys are completed annually in Chad by the Ministry of Public Health along with non-governmental partners to collect key nutrition and mortality indicators. We conducted a secondary data analysis of SMART survey data in the years hemoglobin assessments were included: 2016 to 2019 and 2021. Each year the surveys were administered across all 23 provinces, typically from July to September to capture the ‘lean’ season. The target population included children under 5 years of age (0–59 months), and women of reproductive age (15–49 years). The SMART methodology is a standardized approach that applies a two-stage sampling design to achieve population-representative samples at national and regional levels. Sample size calculations were conducted using the Emergency Nutrition Assessment (ENA) software and approximately 12,000 to 15,000 households were included each year. Detailed sampling methodology can be found in the survey reports [8, 16–19].

Heads of household were administered questionnaires to collect information on socioeconomic factors, such as education level and primary income source and women 15 to 49 years of age were asked about their pregnancy and lactation (PLW) status, receipt of nutritional counseling, and their knowledge of essential nutrition behaviors. For children under 5 years of age, child age (in months), sex, morbidity, receipt of health services, and anthropometric measurements were collected. Additionally, the WHO infant and young child feeding (IYCF) questionnaire was administered to parents of children 6–23 months of age to capture child diet and feeding practices. In half of the sampled households for women and children 6–59 months of age, hemoglobin assessments were conducted using a finger-prick capillary blood draw and Hemocue photometric analyzer (HemoCue Hb301). The full surveys and details on data collection procedures are available in the survey reports [8, 16, 17, 18, 19].

For children 6 to 59 months of age, anemia was defined as hemoglobin less than 11.0 g/dL. For women, anemia was defined as hemoglobin less than 11.0 g/dL and 12.0 g/dL for pregnant women and non-pregnant women, respectively [20]. Hemoglobin values were not adjusted for altitude. IYCF indicators included (1) Minimum dietary diversity (MDD), defined as consumption of ≥ 5 food groups during the previous day; (2) Minimum meal frequency (MMF), defined as breastfed infants receiving two or three feedings of solid, semi-solid, or soft foods for those 6–8 months and 9–23 months respectively, and non-breastfed infants aged 6–23 months receiving four feedings of solid, semi-solid, or soft foods or milk feeds where at least one of the feedings included solid, semi-solid, or soft foods; and (3) Minimum acceptable diet (MAD), defined as achieving both MDD and MMF [21]. Other covariates of interest included agroecological zone and head of household marital status, education level, and primary income source. For women, age, PLW status, receipt of nutrition counseling in the past 3 months, and self-reported knowledge of women’s nutrition and anemia prevention were included; and for children, sex and age were also explored as covariates.

Sampling weights were available for the child data and were applied to the dataset before conducting statistical analyses. The prevalence of anemia in children and women was assessed from 2016 to 2019 and 2021, stratified by agroecological zone and province. Two-sample tests of proportions were conducted to determine whether the differences in anemia prevalence from 2016 to 2021 were statistically significant (p < 0.05). Bivariate logistic regression models were conducted to analyze the relationship between anemia and individual and household characteristics independently for child and women data in 2019 and 2021. Multivariate logistic regression models were created to provide adjusted estimates of odds of anemia for variables that had a statistically significant odds ratio in the bivariate analysis. Additional multivariate logistic regression models were performed to explore the independent mediation of MDD, MMF, and MAD between agroecological zone and the odds of anemia. Analyses were performed using Stata version 15 and R statistical software [22, 23].

## Results

The national and regional trends in anemia in Chad are presented for 2016–2021, excluding 2020 when hemoglobin assessments were not collected due to COVID-19. Risk factors for anemia, including IYCF indicators, are explored for the most recent available data, including 2019 and 2021.

### Trends in anemia, 2016–2021

**Fig. 2:**
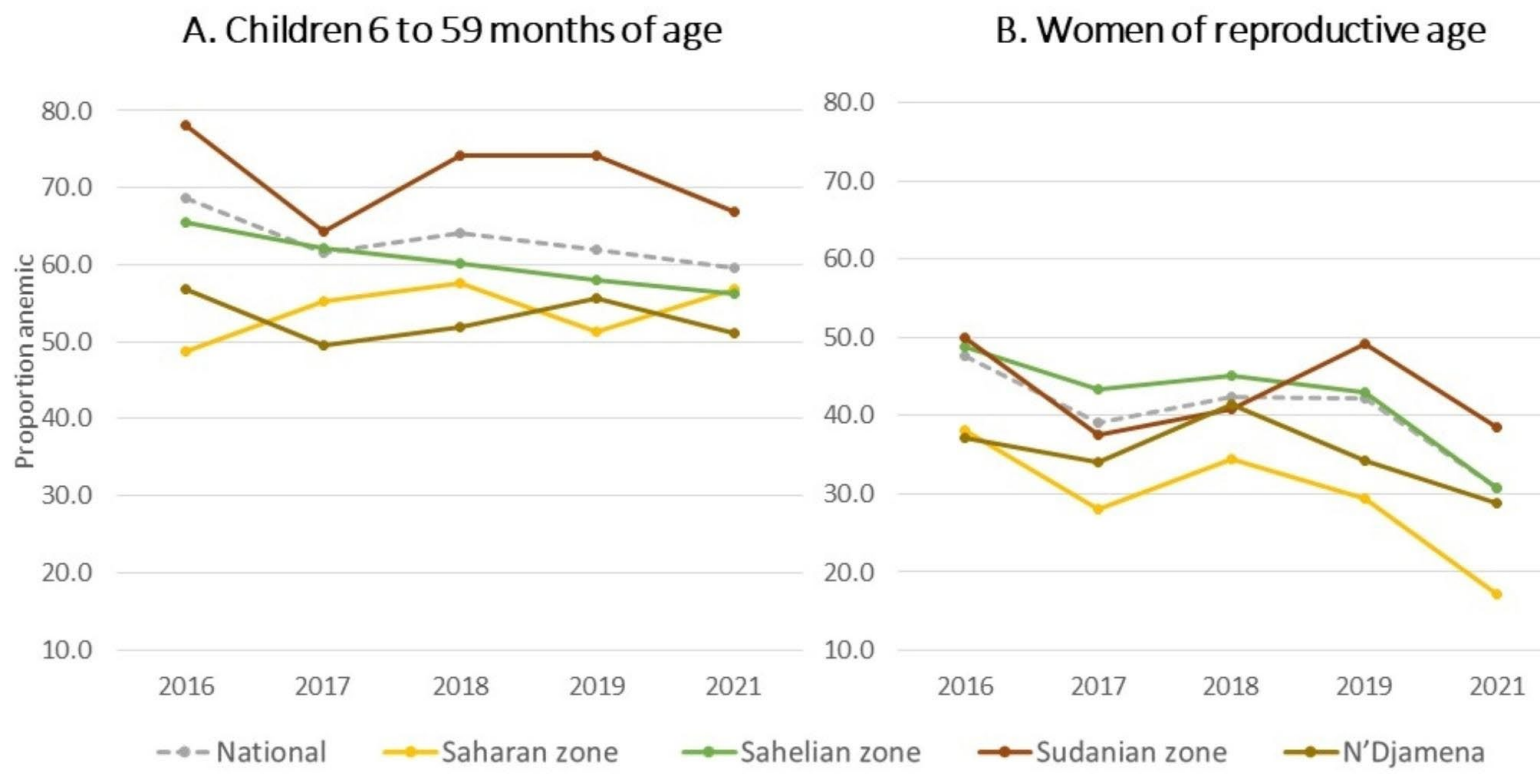
Trends in anemia prevalence in children under five and women of reproductive age by agroecological zone in Chad, 2016–2021

Anemia status was determined from hemoglobin values available from 5,771 to 9,109 children aged 6 to 59 months and 5,214 to 7,198 women 15 to 49 years of age, with variation in the sample size by year. The national prevalence of anemia in children declined from 68.6% to 2016 to 59.6% in 2021 (p < 0.001) (Fig. 2 and Supplementary Tables 1, Additional File 1). The Sudanian zone consistently had the highest prevalence of child anemia from 2016 (78.1%) to 2021 (64.1%), with rates exceeding 80% in the provinces of Logone Occidental, Mandoul, and Mayo Kebi Ouest in 2018 or 2019 (Supplementary Tables 2, Additional File 1). In the Sahelian zone, the prevalence decreased steadily from 65.4% to 2016 to 56.3% in 2021 (p < 0.001). N’Djamena also saw a slight decrease in prevalence of anemia from 56.9% to 2016 to 51.1% in 2021, but this was not statistically significant (p = 0.113). In contrast, between 2016 and 2021 the prevalence in the Saharan zone increased from 48.7 to 56.8% (p = 0.001), with high variability in province-level rates in 2018 and 2019.

The national prevalence of women’s anemia declined significantly from 47.6% to 2016 to 30.8% in 2021 (p < 0.001), with the greatest decreases occurring from 2019 to 2021 (Fig. 2). The Saharan zone had the lowest rates of anemia in women, similar to what was observed for children, and experienced the most significant decline over the six-year period, from 38.2% to 2016 to 17.2% in 2021 (p < 0.001) (Supplementary Tables 1, Additional File 1). The Sahelian and Sudanian zones had the highest rates in the country, where over half the provinces exceeded a prevalence of 50% in 2016, followed by a general decline in 2017, and a resurgence of rates over 50% in several provinces in 2018 and 2019 (Supplementary Tables 3, Additional File 1). However, the two zones had an overall significant decline from 2016 to 2021, from 48.7 to 30.8% (p < 0.001) in the Sahelian zone and 49.9–38.6% (p < 0.001) in the Sudanian zone. In N’Djamena, the anemia prevalence decreased from 37.2% to 2016 to 28.9% in 2021, (p = 0.012).

### Characteristics of children, women, and households in 2019 and 2021

Data were available for 5,879 and 6,636 children 6–59 months of age in 2019 and 2021, respectively. Across both years, age and sex were normally distributed (Table 1). IYCF indicators were lower in 2019 compared to 2021, with 50.6% and 65.3% of children 6–23 months achieving MMF, 7.9% and 12.6% achieving MDD, and 4.7% and 9.3% achieving MAD, respectively (Table 2). Among women of reproductive age, data were available for 5,527 and 6,033 women in 2019 and 2021, respectively. Across both years, age was normally distributed and almost half of women were non-pregnant and non-lactating (49.5% and 49.0%), about a third were lactating only (35.4% and 34.1%), less than a fifth were pregnant only (14.5% and 15.7%), and few were both pregnant and lactating (0.7% and 1.3%) (Table 3). In both years, approximately a fifth (18.0% and 22.9%) of women received nutrition counseling in the previous three months, a quarter reported some knowledge of women’s nutrition (25.2% and 22.9%), and less than 7% reported knowledge of anemia prevention.

**Table 1:** Risk factors for anemia in children under-five in Chad, 2019 and 2021

Characteristics	2019 (n = 5,879)^1^			2021 (n = 6,636)^1^		
Child	n (%)	cOR^2^ (95% CI)	aOR^3^ (95% CI)	n (%)	cOR^2^ (95% CI)	aOR^3^ (95% CI)
Age (in months)						
6–12	764 (13.0)	**3.19 (2.60, 3.93)**	**3.50 (2.83, 4.33)**	848 (12.8)	**5.20 (4.28, 6.35)**	**5.20 (4.26, 6.37)**
12–24	1485 (25.2)	**2.92 (2.46, 3.46)**	**3.05 (2.56, 3.64)**	1572 (23.7)	**4.54 (3.86, 5.35)**	**4.55 (3.86, 5.38)**
24–36	1428 (24.3)	**1.70 (1.44, 2.00)**	**1.71 (1.44, 2.04)**	1698 (25.6)	**2.41 (2.07, 2.81)**	**2.43 (2.08, 2.85)**
36–48	1287 (21.9)	**1.39 (1.17, 1.64)**	**1.43 (1.20, 1.70)**	1698 (21.1)	**1.55 (1.32, 1.81)**	**1.59 (1.35, 1.86)**
48–60	915 (15.6)	1.00	1.00	1117 (16.8)	1.00	1.00
Sex						
Male	3103 (52.7)	1.05 (0.95, 1.17)		3382 (50.1)	0.98 (0.89, 1.08)	
Head of Household						
Sex (male)	4619 (78.6)	0.98 (0.87, 1.12)		5086 (76.6)	1.11 (0.99, 1.25)	
Marital status						
Married	5524 (94.0)	1.00		6148 (92.6)	1.00	1.00
Single/Divorced/ Widowed	355 (6.0)	0.99 (0.80, 1.23)		488 (7.4)	**0.78 (0.65, 0.94)**	0.83 (0.68, 1.01)
Education level						
Illiterate	2828 (48.1)	1.00	1.00	3057 (46.0)	1.00	1.00
Literate	1402 (23.8)	**0.73 (0.64, 0.83)**	**0.76 (0.67, 0.88)**	1577 (23.8)	**0.87 (0.77, 0.99)**	0.93 (0.82, 1.06)
Primary schooling	842 (14.3)	**1.33 (1.13, 1.57)**	0.89 (0.74, 1.08)	938 (14.1)	1.11 (0.95, 1.29)	0.90 (0.76, 1.07)
Secondary/post-secondary	807 (13.7)	1.17 (0.99, 1.38)	0.84 (0.68, 1.03)	1064 (16.1)	1.07 (0.93, 1.23)	0.96 (0.80, 1.15)
Income Source						
Farmer	3792 (64.5)	1.00	1.00	3933 (59.2)	1.00	1.00
Pastoralist	346 (5.9)	**0.52 (0.42, 0.65)**	**0.76 (0.59, 0.98)**	440 (6.6)	0.98 (0.80, 1.02)	1.13 (0.90, 1.41)
Business/transport	783 (13.3)	**0.82 (0.70, 0.96)**	1.09 (0.92, 1.31)	949 (14.3)	**0.73 (0.64, 0.85)**	**0.81 (0.69, 0.96)**
Temporary work	205 (3.5)	0.75 (0.57, 1.00)	0.88 (0.65, 1.20)	247 (3.7)	**0.59 (0.45, 0.76)**	**0.67 (0.51, 0.89)**
Civil servant	274 (4.7)	**0.68 (0.53, 0.87)**	0.89 (0.66, 1.19)	276 (4.2)	**0.60 (0.47, 0.77)**	**0.67 (0.51, 0.89)**
Other salaried work	129 (2.2)	**0.67 (0.48, 0.95)**	0.77 (0.53, 1.13)	202 (3.0)	0.84 (0.63, 1.13)	0.94 (0.69, 1.28)
Unemployed	212 (3.6)	0.99 (0.75, 1.33)	1.20 (0.89, 1.64)	326 (4.9)	0.84 (0.66, 1.05)	0.98 (0.77, 1.26)
Other	138 (2.3)	0.73 (0.52, 1.03)	0.82 (0.57, 1.18)	263 (4.0)	1.02 (0.79, 1.32)	1.13 (0.86, 1.50)
Agroecological Zone						
Saharan zone	887 (15.1)	1.00	1.00	937 (14.1)	1.00	1.00
Sahelian zone	2901 (49.3)	**1.32 (1.13, 1.53)**	**1.25 (1.05, 1.49)**	3194 (48.1)	0.97 (0.84, 1.12)	0.97 (0.82, 1.14)
Sudanian zone	1875 (31.9)	**2.77 (2.34, 3.28)**	**2.86 (2.31, 3.54)**	2184 (32.9)	**1.54 (1.31, 1.80)**	**1.51 (1.24, 1.84)**
N’Djamena	216 (3.7)	1.29 (0.95, 1.74)	1.31 (0.95, 1.82)	321 (4.8)	0.78 (0.61, 1.01)	0.95 (0.71, 1.26)

Legend: *cOR* Crude Odds Ratio; *aOR* Adjusted Odds Ratio; *CI* Confidence Interval; **bold** = statistically significant p-value < 0.05

^1^ Household level data was missing for 124 children in 2019 and 115 children in 2021

^2^ Bivariate logistic regression was performed for each variable independently and results are reported as odds ratio with a 95% confidence interval

^3^ Multivariate logistic regression models were performed for each variable that had a significant OR in the 2019/2021 bivariate analysis. In 2019, child age, head of household education level and income source, and agroecological zone was adjusted for. In 2021, child age, head of household marital status, education level and income source, and agroecological zone were adjusted for

**Table 2:** IYCF indicators and odds of anemia among children 6–23 months in Chad in 2019 and 2021

	2019 (n = 2,170)			2021 (n = 2,349)		
	n (%)	cOR (95% CI)^1^	aOR (95% CI)^2^	n (%)	cOR (95% CI)^1^	aOR (95% CI)^2^
Food Groups						
Vitamin A Rich Foods	398 (18.3)	**1.30 (1.01, 1.69)**	1.24 (0.95, 1.63)	481 (20.5)	**0.66 (0.53, 0.83)**	**0.67 (0.53, 0.84)**
Meat Products	572 (26.4)	0.83 (0.67, 1.03)		724 (30.8)	**0.69 (0.57, 0.84)**	**0.72 (0.58, 0.88)**
Dairy/Eggs	549 (25.3)	**0.71 (0.57, 0.88)**	0.84 (0.67, 1.05)	679 (28.9)	0.90 (0.73, 1.10)	
IYCF Indicators						
MDD	172 (7.9)	**0.66 (0.47, 0.91)**	**0.65 (0.46, 0.92)**	298 (12.6)	**0.63 (0.48, 0.82)**	**0.65 (0.50, 0.86)**
MMF^3^	731 (50.6)	0.87 (0.67, 1.11)		1132 (65.3)	**0.49 (0.38, 0.62)**	**0.49 (0.38, 0.63)**
MAD^4^	91 (4.7)	**0.62 (0.46, 0.85)**	0.69 (0.44, 1.11)	200 (9.3)	**0.62 (0.41, 0.97)**	**0.64 (0.47, 0.89)**

Legend: *cOR* Crude Odds Ratio; *aOR* Adjusted Odds Ratio; *CI* Confidence Interval; ***bold*** = statistically significant p-value < 0.05; *MDD* Minimum Dietary Diversity; *MMF* Minimum Meal Frequency; *MAD* Minimum acceptable diet

^1^ Bivariate logistic regression was performed for each variable independently and results are reported as odds ratio with a 95% confidence interval

^2^ Multivariate logistic regression models were performed for each IYCF variable independently to avoid collinearity and adjusted for potential confounders in 2019 and 2021: child age, head of household education level and income source, and agroecological zone

^3^ MMF was missing for 723 and 616 children in 2019 and 2021, respectively

^4^ MAD was missing for 265 and 213 children in 2019 and 2021, respectively

**Table 3:** Risk factors for women’s anemia in Chad in 2019 and 2021

Characteristics	2019 (n = 5,527)			2021 (n = 6,033)		
Woman	n (%)	cOR (95% CI)^1^	aOR (95% CI)^2^	n (%)	cOR (95% CI)	aOR (95% CI)^2^
Age						
15–19	1,029 (18.6)	1.06 (0.91, 1.23)		1,186 (19.7)	1.14 (0.98, 1.32)	
20–29	2,310 (41.8)	1.00		2,453 (40.7)	1.00	
30–39	1,540 (27.9)	0.94 (0.82, 1.07)		1,665 (27.6)	1.01 (0.89, 1.16)	
40–49	648 (11.7)	1.02 (0.85, 1.21)		729 (12.1)	1.01 (0.84, 1.20)	
Status^3^						
Pregnant	801 (14.5)	0.95 (0.81, 1.12)		944 (15.7)	0.94 (0.80, 1.10)	1.02 (0.86, 1.20)
Lactating	1,954 (35.4)	1.10 (0.98, 1.24)		2,055 (34.1)	**1.12 (1.00, 1.26)**	1.13 (0.99, 1.27)
Pregnant & lactating	37 (0.7)	0.59 (0.29, 1.21)		80 (1.3)	**0.32 (0.17, 0.63)**	**0.31 (0.16, 0.61)**
NPNL	2,735 (49.5)	1.00		2,954 (49.0)	1.00	1.00
Nutrition counseling^4^						
Receipt in last 3 mo.	994 (18.0)	**0.71 (0.62, 0.82)**	**0.68 (0.59, 0.79)**	1,378 (22.9)	0.92 (0.81, 1.05)	
Nutrition knowledge						
Women’s nutrition	1,391 (25.2)	1.01 (0.89, 1.14)		1,639 (27.2)	**0.86 (0.76, 0.97)**	**0.78 (0.69, 0.89)**
Anemia prevention	297 (5.4)	1.06 (0.83, 1.34)		417 (6.9)	0.86 (0.69, 1.07)	
Head of Household						
Sex (male)	4,124 (74.6)	0.96 (0.85, 1.08)		4,364 (72.3)	0.96 (0.85, 1.09)	
Marital status						
Married	5,013 (90.7)	1.00		5,383 (89.2)	1.00	
Single/Divorced/ Widowed	514 (9.3)	0.96 (0.80, 1.15)		650 (10.8)	1.06 (0.89, 1.27)	
Education level						
Illiterate	2,848 (51.5)	1.00	1.00	2,939 (48.7)	1.00	1.00
Literate	1,213 (22.0)	**0.73 (0.63, 0.84)**	**0.75 (0.65, 0.87)**	1,440 (23.9)	**0.83 (0.72, 0.95)**	**0.81 (0.70, 0.93)**
Primary schooling	706 (12.8)	1.05 (0.89, 1.24)	0.94 (0.79, 1.12)	792 (13.1)	**1.32 (1.11, 1.55)**	1.07 (0.90, 1.28)
Secondary/post-secondary	760 (13.8)	0.92 (0.78, 1.08)	0.88 (0.73, 1.06)	862 (14.3)	1.09 (0.93, 1.28)	0.91 (0.75, 1.09)
Income Source						
Farmer	3,426 (62.0)	1.00	1.00	3,380 (56.0)	1.00	1.00
Pastoralist	346 (6.3)	**0.68 (0.54, 0.86)**	0.81 (0.63, 1.02)	429 (7.1)	**0.71 (0.57, 0.89)**	0.92 (0.73, 1.17)
Business/transport	756 (13.7)	**0.63 (0.54, 0.75)**	**0.69 (0.59, 0.82)**	981 (16.3)	**0.58 (0.49, 0.68)**	**0.64 (0.54, 0.75)**
Temporary work	220 (4.0)	0.77 (0.58, 1.02)	0.79 (0.60, 1.05)	232 (3.9)	**0.57 (0.41, 0.78)**	**0.55 (0.40, 0.76)**
Civil servant	278 (5.0)	**0.64 (0.50, 0.83)**	**0.69 (0.52, 0.90)**	266 (4.4)	**0.72 (0.55, 0.95)**	0.76 (0.56, 1.01)
Other salaried work	117 (2.1)	**0.66 (0.45, 0.97)**	0.69 (0.47, 1.02)	186 (3.1)	0.93 (0.68, 1.28)	0.85 (0.62, 1.17)
Unemployed	249 (4.5)	**0.63 (0.48, 0.83)**	**0.66 (0.51, 0.87)**	345 (5.7)	**0.61 (0.47, 0.79)**	**0.64 (0.50, 0.83)**
Other	135 (2.4)	0.79 (0.55, 1.12)	0.81 (0.57, 1.16)	214 (3.6)	0.83 (0.62, 1.12)	0.88 (0.65, 1.19)
Agroecological Zone						
Saharan zone	862 (15.6)	1.00	1.00	995 (16.5)	1.00	1.00
Sahelian zone	2,770 (50.1)	**1.81 (1.54, 2.14)**	**1.80 (1.52, 2.14)**	2,885 (47.8)	**2.17 (1.89, 2.60)**	**2.13 (1.77, 2.57)**
Sudanian zone	1,636 (29.6)	**2.27 (1.91, 2.71)**	**2.42 (2.00, 2.94)**	1,817 (30.1)	**3.06 (2.53, 3.69)**	**3.16 (2.57, 3.89)**
N’Djamena	259 (4.7)	1.19 (0.88, 1.60)	**1.46 (1.07, 1.98)**	336 (5.6)	**1.98 (1.49, 2.64)**	**2.27 (1.68, 3.06)**

Legend: *cOR* Crude Odds Ratio; *aOR* Adjusted Odds Ratio; *CI* Confidence Interval; **bold** = statistically significant p-value < 0.05; *NPNL* Non-pregnant non-lactating

^1^ Bivariate logistic regression was performed for each variable independently and results are reported as odds ratio with a 95% confidence interval

^2^ Multivariate logistic regression models were performed for each variable that had a significant OR in the 2019/2021 bivariate analysis. In 2019, nutrition counseling, education level and income source of the head of household, and agroecological zone were adjusted for. In 2021, woman status, nutrition knowledge, education level and income source of the head of household, and agroecological zone were adjusted for

^3^ 70 and 93 observations were missing for Woman Status in 2019 and 2021, respectively

^4^ 40 and 21 observations were missing for Nutrition Counseling in 2019 and 2021, respectively

Household characteristics of the sampled children and women in 2019 and 2021 are presented in Tables 1 and 3, respectively, and the distributions are similar. Most households were male headed (72.3–78.6%) and the majority were married (89.2–94.0%). Approximately half of household heads were illiterate (46.0-51.5%) and the most common primary income source was farming (56.0-64.5%). About half of households resided in the Sahelian zone, less than one-third in the Sudanian zone, ~ 15% in the Saharan zone, and ~ 5% in N’Djamena.

### Determinants of anemia in children 6–59 months of age, 2019 and 2021

In both years, there was a linear relationship between age and the odds of anemia, whereby younger children had greater odds of being anemic than older children (Table 1). In 2019, children under 24 months were over three times as likely to be anemic compared to children 48–60 months after adjusting for other variables (6–12 months AOR: 3.50, 95%CI: 2.83, 4.33; 12–24 months AOR:3.05, 95%CI: 2.56, 3.64). In 2021, children 6–12 and 12–24 months were 5.20 times (95%CI: 4.26, 6.37) and 4.55 times (95%CI:3.86, 5.38) as likely to be anemic compared to children 48–60 months even after adjusting for other significant variables. Child sex, marital status and sex of the head of the household were not significantly associated with child anemia in 2019 or 2021. In 2019 and 2021, children had significantly lower odds of anemia with a head of household who reported being literate compared to illiterate; in adjusted models, this finding remained significant only in 2019 (AOR: 0.76, CI: 0.67, 0.88). In 2019, children of pastoralists had lower odds of anemia, (AOR: 0.76 95%CI: 0.59, 0.98), whereas in 2021, children of business/transport workers (AOR: 0.81 95%CI: 0.69, 0.96), civil servants (AOR: 0.67 95%CI: 0.51, 0.89), and temporary workers (AOR: 0.67 95%CI: 0.51, 0.89) had lower odds of anemia compared to children of farmers.

Agroecological zone was significantly associated with child anemia in 2019 and 2021, though to varying degrees. In the 2019 adjusted models, the odds ratio for children in the Sudanian zone was 2.86 (95%CI: 2.31, 3.54), making them almost three times as likely to be anemic compared to children in the Saharan zone. Children in the Sahelian zone also had a significantly higher likelihood of having anemia, with an odds ratio of 1.32 (95%CI: 1.05, 1.49). In 2021, after adjusting for the other significant variables, children in the Sudanian zone were still more likely to have anemia than children in the Saharan zone but differences were less pronounced (AOR: 1.51, 95%CI:1.24, 1.84). The odds of anemia in the Sahelian zone were not significantly different from the Saharan zone in 2021 and N’Djamena rates were similar to the Saharan zone both years.

### IYCF and the relationship to anemia among children 6–23 months of age, 2019 and 2021

The relationship between consumption of select food groups and anemia in children differed in 2019 and 2021 (Table 2). In 2019, children who consumed vitamin A rich foods exhibited higher odds of anemia, but this relationship was non-significant in the adjusted model (AOR: 1.24, 95%CI: 0.95, 1.63). In 2021, children who consumed vitamin A rich foods had significantly lower odds of anemia than children who did not (AOR: 0.67, 95%CI: 0.53, 0.84). Consumption of meat products was not associated with anemia in 2019 but was associated with reduced odds of anemia in 2021 (AOR: 0.72, 95%CI: 0.58, 0.88). Consumption of dairy/eggs was not associated with reduced odds of anemia after adjusting for individual and household factors. In both 2019 and 2021, achieving MDD was associated with 35% reduced odds of anemia in the adjusted models (95%CI: 0.46, 0.92; 95%CI: 0.50, 0.86). In 2021, children who achieved MMF or MAD also had significantly reduced odds of anemia (AOR: 0.49, 95%CI: 0.38, 0.63; AOR: 0.64, 95%CI: 0.47, 0.89) compared to children who did not. However, in 2019, MMF and MAD were not associated with the odds of anemia after adjusting for other significant variables.

Table 4 presents the odds ratio of anemia in children by agroecological zone, both unadjusted and adjusted for MDD, MMF, and MAD independently. In 2019, the unadjusted odds of anemia were 1.32 times and 2.77 times greater in the Sahelian and Sudanian zones as compared to the Saharan zone. These were not attenuated by the addition of any one of the three IYCF indicators, and the AORs between agroecological zone and anemia remained similar. In N’Djamena, the addition of any of the IYCF variables identified a more acute relationship, whereby the odds of anemia were about twice as high in the capital than in the Saharan zone, as compared to the unadjusted OR of 1.29 (95%CI: 0.95, 1.74). In 2021, a different pattern was observed where the addition of any one of the IYCF indicators to the model appeared to mediate the relationship between agroecological zone and anemia, specifically in the Sudanian context. With the addition of MDD, MMF, or MAD in the models, the odds of anemia went from 1.54 (95%CI: 1.31, 1.80) to 1.04–1.25, all non-significant.

**Table 4:** Odds of anemia by agroecological zone after adjusting for child diet among children 6–23 months in Chad, 2019–2021

	2019 (n = 2,170)				2021 (n = 2,349)			
Agroecological Zone	cOR (95% CI)^1^	Adjusted for MDD^2^	Adjusted for MMF	Adjusted for MAD	cOR (95%CI)	Adjusted for MDD	Adjusted for MMF	Adjusted for MAD
Saharan zone	1.00	1.00	1.00	1.00	1.00	1.00	1.00	1.00
Sahelian zone	**1.32** **(1.13, 1.53)**	**1.58** **(1.20, 2.06)**	**1.61** **(1.12, 2.28)**	**1.70** **(1.28, 2.25)**	0.97 (0.84, 1.12)	1.14 (0.86, 1.52)	1.02 (0.71, 1.46)	1.17 (0.87, 1.58)
Sudanian zone	**2.77** **(2.34, 3.28)**	**2.25** **(1.66, 3.04)**	**2.22** **(1.52, 3.22)**	**2.44** **(1.78, 3.34)**	**1.54** **(1.31, 1.80)**	1.25 (0.93, 1.67)	1.04 (0.71, 1.48)	1.22 (0.90, 1.66)
N’Djamena	1.29 (0.95, 1.74)	**1.86** **(1.10, 3.23)**	**2.09** **(1.08, 4.28)**	**2.23** **(1.26, 4.14)**	0.78 (0.61, 1.01)	0.68 (0.42, 1.11)	0.61 (0.34, 1.14)	0.72 (0.43, 1.21)

Legend: *cOR* Crude Odds Ratio; *CI* Confidence Interval; ***bold*** = statistically significant p-value < 0.05; *MDD* Minimum Dietary Diversity; *MMF* Minimum Meal Frequency; *MAD* Minimum acceptable diet

^1^ Bivariate logistic regression was performed for agroecological zone and results are reported as odds ratio with a 95% confidence interval

^2^ Multivariate logistic regression models were performed for each IYCF variable independently and results are reported as odds ratios with a 95% confidence interval

### Determinants of women’s anemia in 2019 and 2021

Differences were observed in the factors associated with the odds of anemia in women in 2019 and 2021 (Table 3). In 2019, women’s receipt of nutrition counseling in the last 3 months was associated with reduced odds of anemia in the unadjusted and adjusted models (AOR: 0.68, 95%CI: 0.59, 0.79), but this was not seen in 2021. PLW status was not associated with anemia in 2019, but in 2021, women who were both pregnant and lactating had reduced odds of anemia compared to those NPNL (AOR: 0.31, 95%CI: 0.16, 0.61), though the sample size was small (n = 80). In 2021, women who reported knowledge of women’s nutrition had reduced odds of anemia (AOR: 0.78, 95%CI: 0.69, 0.89), but the same was not true for those that reported specific knowledge of anemia prevention (OR: 0.86, 95%CI: 0.69, 1.07). Report of any nutrition knowledge was not associated with anemia in 2019. Age was not associated with anemia in either year.

In both years, women who lived in a household where the head of household was literate had lower odds of anemia compared to illiterate household heads (AOR: 0.75–0.81). Women living in households where the primary income source was farming had greater odds of anemia than any of the other occupations, though not always significant. In 2019, households with income sources from business/transport, civil service, and no employment had significantly lower odds of anemia in women after adjusting for other variables, compared to farmers. In 2021, business/transport, temporary work, and the unemployed were less likely to have anemia than those working as farmers (Table 3). Marital status and sex of head of household were not associated with women’s anemia.

In both 2019 and 2021, agroecological zone was a significant predictor of women’s anemia. Women living in the Sudanian zone were 2.41 (95%CI: 2.00, 2.94) and 3.16 (95%CI: 2.57, 3.89) times more likely to have anemia as compared to women living in the Saharan zone in 2019 and 2021, respectively. In 2019, women living in the Sahelian zone and N’Djamena also experienced 1.80 (95% CI: 1.52, 2.14) and 1.46 (95%CI: 1.07, 1.98) times greater odds of anemia compared to those in the Sahara, after adjusting for other significant variables. The same trend was found in 2021, with even greater odds ratios between any of the other regions and the Saharan zone (Table 3).

## Discussion

From 2016 to 2021, there was a significantly declining trend in anemia in Chad with a 9.0% reduction among children 6–59 months of age and a 16.8% decline to 30.8% among women of reproductive age. Despite the observed decline, a 59.6% prevalence of anemia in children continues to be a severe public health problem in Chad, particularly in the Sudanian zone. Across the five years of data, the anemia prevalence in the Sudanian zone was consistently the highest and exceeded 80% in the provinces of Logone Occidental, Mandoul, and Mayo Kebi Ouest in some years. This finding was surprising given that the Sudanian zone is generally regarded as more food secure and economically stable. Recent analyses have demonstrated that between 2016 and 2021, households in the Sudanian zone consistently had higher food consumption scores than those in the Saharan and Sahelian zones, and households in the Sudanian zone were less likely to have COVID-19 income losses as compared to those in the other agro-ecological zones [24, 25]. Additionally, children 6–23 months of age in the Sudanian zone had lower odds of wasting as compared to the Sahelian and Saharan zones, even after adjusting for child diet [26]. In this analysis, we observed that adjusting for MDD, MMF, or MAD in 2019 slightly decreased the odds of anemia in the Sudanian zone and, in 2021, attenuated the odds of anemia such that no significant difference was perceivable when compared to the Saharan zone. These findings suggest that despite lower rates of acute malnutrition and related macronutrient deficiencies, young children in the Sudanian zone may be lacking micronutrient rich diets. In line with this finding, we found that children under 2 years of age who achieved MDD had 35% lower odds of anemia in 2019 and 2021 in the country-wide samples. However, the proportion of children who achieved MDD was very low – only 7.9% in 2019 and 12.6% in 2021. Improvements in infant feeding practices, with a focus on diet diversity and the inclusion of iron and micronutrient-rich foods, should be promoted for the prevention of anemia in all regions of Chad.

Another possible cause of high anemia prevalence in the Sudanian zone is helminth infections. Intestinal helminths may cause anemia through reduced food intake and malabsorption, with hookworm and schistosomes being the most prevalent anemia-causing intestinal helminths in Chad. Among school age children, Hookworm was most prevalent in the Sudan (44%) and tropical zones (76%), both of which are included in the Sudanian zone in this analysis [27]. Areas with higher prevalence of helminth infection are relatively well aligned with high levels of child anemia, indicating that preventative chemotherapy (deworming) programs, as recommended by the WHO [28], may contribute to anemia reduction in the Sudanian zone.

From 2019 to 2021, both women and children experienced declines in anemia in the Sudanian and Sahelian zones, along with N’Djamena. However, in the Saharan zone, the rate increased among children whereas it decreased for women. This increase in child anemia in the Saharan zone is likely responsible in part for the differences seen in the odds of anemia by zone between 2019 and 2021, where only the Sudanian zone maintained significantly higher odds in 2021 when compared to the Saharan zone. In contrast, we observed higher odds of women’s anemia in 2021 compared to 2019, as the rate in the Saharan zone continued to decline and the differences across zones were similar in 2019 and 2021. This discrepancy in trends between women and children could be a subject of further research in the Saharan zone but is likely linked to poor maternal and child nutrition practices and the association between livestock ownership, which is higher in the Saharan zone, and zoonotic / sanitation related diseases to which younger children are more susceptible [29]. While diets in the Saharan zone may be generally higher in iron rich foods, pastoralists are also subject to food insecurity in the face of climatic hazards, intercommunal conflict, or both. There was a peak in food insecurity in 2020 in the Saharan zone, where 46% had poor or borderline food consumption and 58% were using extreme coping measures [25], which may have contributed to the increases in anemia among children in the Saharan zone in 2021, though this does not explain the decrease seen in women’s anemia.

Among children and women, higher odds of anemia were present in households in which the head of household was illiterate, and the primary income source was farming. These differences highlight how inequities in socioeconomic condition are contributors to poor nutritional status in Chad. Targeted approaches to address these inequities should be adopted in future national nutrition programming. The greater odds of anemia in children under two years of age compared to older children indicates a need for improved infant and young child feeding practices that promote anemia reduction in young infants. While no differences were observed in the odds of anemia between pregnant and NPNL women, the high rates of anemia in pregnancy – 40% in 2019 and 29% in 2021 – suggests that deficiencies may be starting in utero. Adherence to iron supplementation during pregnancy in Chad is estimated at only 12% [30]. Reduced odds of anemia in women who reported receiving nutrition counseling in the past 3 months in 2019, and in women who reported knowledge of essential nutrition actions for women’s nutrition in 2021, indicate the potential for greater improvements in anemia reduction with comprehensive nutrition counseling for women of reproductive age. Continued efforts to improve coverage of nutrition education and iron folic acid or multiple micronutrient supplementation in pregnancy is key to reduce anemia rates in both mothers and young infants.

Our analysis has some important limitations. Due to the nature of the data, it was not possible to link mother and child pairs and analyze the role of maternal factors on child anemia. It was also not possible to adjust hemoglobin levels for altitude, which could result in an underestimation of anemia in the Tibesti mountain range. Additionally, the SMART surveys did not contain information on the diets of women or children older than 24 months. Therefore, the contribution of dietary deficiencies to the prevalence of anemia in Chad could not be explored in these groups. Similarly, information on other known risk factors for anemia present in Chad, including malaria, sickle cell disease, and other infectious diseases, were not available in this dataset. Despite this, our study has significant strengths. This analysis utilized a nationally representative sample that summarizes six years of SMART survey data to reveal national and regional trends in anemia among both children and women of reproductive age. Agroecological zone was identified as an important predictor of anemia, highlighting the need for a targeted approach to address anemia in Chad. Other important determinants, such as child age, infant feeding practices, household socioeconomic conditions, and maternal receipt of nutrition counseling, can further inform future policies and programming for anemia reduction in Chad.

## Conclusions

The national prevalence of anemia among children and women was 60% and 31% in 2021, respectively, with the highest prevalence rates observed in the Sudanian zone. Anemia prevalence declined significantly in both populations from 2016 to 2021, however, this trend differed by agro-ecological zone. High anemia prevalence rates, which are coupled with high levels of food insecurity and chronic malnutrition, require additional attention. Apart from strategies to enhance food access and consumption nationally, targeted efforts to reduce anemia in the Sudanian zone, where prevalence was the highest, and in the Saharan zone, where anemia prevalence increased, are needed. Across zones, children under two years of age consistently had higher rates of anemia as compared to older children, and achievement of minimum dietary diversity was associated with a significant reduction in anemia in children less than 24 months, stressing the need for interventions to improve IYCF practices nationally. Household head literacy and women’s nutrition and anemia knowledge were associated with reduced anemia risk, suggesting that education interventions may be effective in reducing anemia. Additionally, increasing the coverage of iron folic acid supplementation in pregnancy, malaria prevention and treatment, and deworming interventions could help to lower the prevalence of anemia in women and children.

### Electronic supplementary material

Below is the link to the electronic supplementary material.

Supplementary Material 1

## Data Availability

The data analyzed during the current study are not publicly accessible but available upon reasonable request from World Food Programme, Chad.

## Abbreviations

SMART: Standardized Monitoring and Assessment of Relief and Transitions
ENA: Emergency Nutrition Assessment
PLW: Pregnant Lactating Woman
WHO: World Health Organization
IYCF: Infant and Young Child Feeding
MDD: Minimum Dietary Diversity
MMF: Minimum Meal Frequency
MAD: Minimum acceptable diet
AOR: Adjusted Odds Ratio
CI: Confidence Interval
