# Supplementary material for: Trends and determinants of anemia in children 6–59 months and women of reproductive age in Chad from 2016 to 2021
Source: BMC Nutr. 2023 Oct 23;9:117. doi: 10.1186/s40795-023-00777-y (PMC10594667; doi:10.1186/s40795-023-00777-y)
Supplement: Supplementary file 1 — Supplementary Material 1 [file 40795_2023_777_MOESM1_ESM.docx]

## Additional File 1

Supplementary Table 1. National and regional trends in the prevalence of anemia in children under-five and women of reproductive age (15-49) in Chad, 2016-2021

|  | **2016** | | **2017** | | **2018** | | **2019** | | **2021** | | **Test of proportions** |
| --- | --- | --- | --- | --- | --- | --- | --- | --- | --- | --- | --- |
|  | **n** | **% (95% CI)** | **n** | **% (95% CI)** | **n** | **% (95% CI)** | **n** | **% (95% CI)** | **n** | **% (95% CI)** |  |
| **Children** |  |  |  |  |  |  |  |  |  |  |  |
| National | 9,109 | 68.6 (67.7, 69.6) | 6,932 | 61.6 (60.5, 62.8) | 5,771 | 64.2 (63.0, 65.5) | 6,003 | 62.0 (60.8, 63.3) | 6,751 | 59.6 (58.5, 60.8) | **0.000** |
| Saharan zone | 744 | 48.7 (45.1, 52.2) | 646 | 55.3 (51.4, 59.1) | 510 | 57.6 (53.4, 61.9) | 924 | 51.4 (48.2, 54.6) | 962 | 56.8 (53.6, 59.9) | **0.001** |
| Sahelian zone | 4,313 | 65.4 (63.9, 66.8) | 3,536 | 62.1 (60.5, 63.7) | 3,040 | 60.2 (58.5, 62.0) | 2,940 | 58.0 (56.2, 59.8) | 3,233 | 56.3 (54.6, 58.0) | **0.000** |
| Sudanian zone | 3,623 | 78.1 (76.7, 79.4) | 2,417 | 64.3 (62.4, 66.2) | 1,930 | 74.1 (72.1, 76.0) | 1,914 | 74.1 (72.2, 76.1) | 2,229 | 64.1 (62.1, 66.1) | **0.000** |
| N’Djamena | 429 | 56.9 (52.2, 61.6) | 333 | 49.5 (44.2, 54.9) | 291 | 51.9 (46.1, 57.6) | 225 | 55.6 (49.1, 62.0) | 327 | 51.1 (45.7, 56.5) | 0.113 |
| **Women** |  |  |  |  |  |  |  |  |  |  |  |
| National | 7,198 | 47.6 (46.4, 48.7) | 6,082 | 39.1 (37.9, 40.4) | 5,214 | 42.3 (41.0, 43.7) | 5,527 | 42.2 (41.0, 43.5) | 6,033 | 30.8 (29.7, 32.0) | **0.000** |
| Saharan zone | 482 | 38.2 (33.9, 42.6) | 616 | 28.1 (24.7, 31.8) | 585 | 34.4 (30.6, 38.3) | 862 | 29.3 (26.4, 32.3) | 995 | 17.2 (15.1, 19.7) | **0.000** |
| Sahelian zone | 3,420 | 48.7 (47.0, 50.3) | 2,922 | 43.4 (41.6, 45.2) | 2,656 | 45.1 (43.2, 47.0) | 2,770 | 42.9 (41.1, 44.8) | 2,885 | 30.8 (29.2, 32.5) | **0.000** |
| Sudanian zone | 2,774 | 49.9 (48.0, 51.7) | 2,109 | 37.5 (35.5, 40.0) | 1,637 | 40.9 (38.5, 43.3) | 1,636 | 49.1 (46.8, 51.5) | 1,817 | 38.6 (36.4, 40.8) | **0.000** |
| N’Djamena | 522 | 37.2 (33.1, 41.4) | 435 | 34.0 (29.7, 38.6) | 336 | 41.4 (36.2, 46.7) | 259 | 34.2 (28.8, 39.9) | 336 | 28.9 (24.4, 33.9) | **0.012** |

Legend:

^1^ A two sample test of proportions, weighted for the child data, was performed to assess whether there was a significant difference in the prevalence of anemia in children 6-59 months and in women 15-49 years of age between 2016 and 2021. Significant p-values are bolded. CI; confidence interval.

Supplementary Table 2. Province-level trends in the prevalence of anemia in children under-five in Chad, 2016-2021

|  | **2016** | **2017** | **2018** | **2019** | **2021** |
| --- | --- | --- | --- | --- | --- |
|  | % (95% CI) | % (95% CI) | % (95% CI) | % (95% CI) | % (95% CI) |
| National | 68.6 (67.7, 69.6) | 61.6 (60.5, 62.8) | 64.2 (63.0, 65.5) | 62.0 (60.8, 63.3) | 59.6 (58.5, 60.8) |
| Tibesti | 50.6 (43.2, 58.0) | 51.8 (43.5, 60.0) | 71.0 (55.0, 86.9) | 32.2 (25.8, 38.6) | 62.6 (55.9, 69.4) |
| Borkou | 51.1 (43.9, 58.2) | 54.0 (44.2, 63.8) | 82.5 (76.8, 88.3) | 46.7 (40.5, 53.0) | 53.6 (46.6, 60.7) |
| Ennedi Est | 47.9 (41.3, 54.6) | 58.5 (52.0, 65.1) | 41.3 (33.2, 49.3) | 62.1 (55.8, 68.4) | 53.4 (48.2, 58.6) |
| Ennedi Ouest | 44.7 (36.9, 52.4) | 54.8 (47.7, 61.9) | 44.7 (37.2, 52.2) | 62.2 (56.1, 68.3) | 59.6 (53.2, 66.3) |
| Ouaddai | 56.0 (50.2, 61.9) | 64.3 (58.1, 70.5) | 40.3 (34.4, 46.2) | 45.4 (40.2, 50.6) | 50.3 (44.7, 55.9) |
| Wadi Fira | 53.6 (49.2, 58.0) | 54.4 (49.0, 59.7) | 39.2 (33.1, 45.3) | 45.3 (40.5, 50.1) | 57.6 (51.9, 63.3) |
| Salamat | 67.7 (63.4, 71.9) | 63.2 (57.9, 68.5) | 59.6 (53.3, 65.8) | 65.3 (60.3, 70.3) | 58.1 (52.3, 63.9) |
| Sila | 57.7 (53.3, 62.1) | 57.8 (52.4, 63.3) | 59.9 (54.2, 65.6) | 67.1 (61.8, 72.5) | 42.0 (36.5, 47.5) |
| Batha | 67.6 (62.8, 72.4) | 65.3 (60.3, 70.3) | 73.0 (68.9, 77.2) | 52.2 (46.0, 58.3) | 58.1 (52.0, 64.3) |
| Guera | 58.8 (52.7, 64.8) | 57.0 (50.9, 63.0) | 64.2 (58.9, 69.4) | 50.5 (43.8, 57.2) | 63.3 (57.2, 69.4) |
| Kanem | 67.2 (62.3, 72.2) | 60.9 (55.5, 66.2) | 63.7 (57.6, 69.8) | 69.9 (62.9, 77.0) | 55.3 (49.8, 60.9) |
| Barh El-Gazel | 65.6 (60.8, 70.3) | 60.8 (54.9, 66.7) | 59.1 (53.4, 64.8) | 72.7 (67.0, 78.3) | 55.5 (50.1, 60.8) |
| Lac | 71.4 (67.1, 75.7) | 62.6 (58.5, 66.7) | 65.1 (59.1, 71.0) | 64.4 (57.5, 712) | 60.1 (54.2, 65.9) |
| Hadjer Lamis | 73.4 (68.9, 78.0) | 60.8 (55.7, 65.9) | 65.5 (59.8, 71.3) | 63.4 (57.7, 69.2) | 60.2 (55.0, 65.4) |
| Chari Baguirmi | 77.7 (73.9, 81.6) | 76.3, 71.4, 81.2) | 64.0 (57.7, 70.3) | 55.0 (48.3, 61.8) | 61.1 (55.7, 66.4) |
| Mayo Kebi Est | 79.5 (75.5, 83.6) | 70.3 (65.6, 75.0) | 66.8 (60.4, 73.2) | 65.6 (59.9, 71.4) | 65.1 (59.7, 70.5) |
| Mayo Kebi Ouest | 75.5 (71.2, 79.8) | 75.0 (70.8, 79.2) | 81.3 (77.3, 85.4) | 79.9 (74.7, 85.1) | 76.0 (71.6, 80.5) |
| Tandjile | 79.4 (76.5, 82.4) | 67.3 (32.3, 72.3) | 65.2 (59.6, 70.8) | 69.9 (64.8, 75.0) | 77.0 (72.6, 81.3) |
| Moyen Chari | 79.6 (75.4, 83.7) | 63.2 (58.1, 68.4) | 65.4 (59.3, 71.5) | 69.5 (62.9, 76.1) | 52.4 (47.3, 57.5) |
| Mandoul | 74.7 (71.4, 78.0) | 55.5 (50.2, 60.8) | 66.2 (60.5, 71.8) | 87.0 (82.1, 92.0) | 50.6 (44.6, 56.5) |
| Logone Occidental | 80.9 (77.5, 84.3) | 57.1 (52.2, 62.1) | 88.1 (84.4, 91.8) | 74.7 (70.4, 79.0) | 68.8 (63.2, 74.4) |
| Logone Oriental | 77.5 (74.2, 80.9) | 58.7 (52.7, 64.7) | 79.2 (74.4, 83.9) | 75.8 (71.3, 80.3) | 76.8 (72.1, 81.5) |
| N’Djamena | 56.9 (52.2, 61.6) | 49.5 (44.2, 54.9) | 51.9 (46.1, 57.6) | 55.6 (49.1, 62.0) | 51.1 (45.7, 56.5) |

CI; confidence interval

Supplementary Table 3. Province-level trends in the prevalence of anemia in women of reproductive age in Chad, 2016-2021

|  | 2016 | 2017 | 2018 | 2019 | 2021 |
| --- | --- | --- | --- | --- | --- |
|  | % (95% CI) | % (95% CI) | % (95% CI) | % (95% CI) | % (95% CI) |
| National | 47.6 (46.4, 48.7) | 39.1 (37.9, 40.4) | 42.3 (41.0, 43.7) | 42.2 (41.0, 43.5) | 30.8 (29.7, 32.0) |
| Tibesti | 23.9 (16.3, 33.7) | 15.3 (10.3, 22.1) | 35.7 (24.3, 49.0) | 19.4 (14.4, 25.5) | 14.4 (10.3, 19.8) |
| Borkou | 43.0 (35.4, 51.1) | 21.1 (14.5, 29.5) | 32.6 (26.1, 39.0) | 28.1 (22.9, 33.9) | 18.6 (14.2, 24.1) |
| Ennedi Est | 26.4 (19.4, 35.0) | 35.9 (29.5, 43.0) | 31.0 (24.5, 38.3) | 31.4 (25.6, 37.8) | 20.2 (16.2, 24.8) |
| Ennedi Ouest | 55.1 (46.0, 63.8) | 34.9 (28.1, 42.5) | 38.9 (32.0, 46.2) | 36.9 (30.9, 43.2) | 14.5 (10.7, 19.4) |
| Ouaddai | 38.4 (32.2, 45.1) | 44.8 (37.7, 52.1) | 21.9 (17.2, 27.5) | 23.8 (19.2, 29.2) | 24.4 (19.7, 29.7) |
| Wadi Fira | 31.9 (27.2, 37.0) | 34.1 (28.6, 40.0) | 20.7 (16.0, 26.4) | 25.4 (20.9, 30.5) | 20.2 (15.9, 25.4) |
| Salamat | 41.8 (36.9, 47.0) | 43.7 (38.0, 50.0) | 58.8 (51.7, 65.5) | 48.5 (43.4, 53.6) | 38.1 (32.0, 44.7) |
| Sila | 37.9 (33.2, 42.9) | 42.3 (36.7, 48.1) | 58.0 (51.5, 64.2) | 44.5 (39.3, 50.0) | 20.8 (16.5, 26.0) |
| Batha | 49.3 (43.5, 55.1) | 46.0 (40.3, 51.9) | 40.8 (35.6, 46.2) | 40.8 (34.5, 47.4) | 29.6 (24.2, 35.7) |
| Guera | 55.2 (48.6, 59.6) | 47.5 (41.0, 54.1) | 38.8 (33.3, 44.7) | 36.7 (29.9, 44.1) | 30.0 (24.4, 36.2) |
| Kanem | 54.2 (48.6, 59.6) | 45.1 (39.3, 51.1) | 51.9 (45.2, 58.5) | 54.2 (47.3, 61.0) | 27.5 (22.3, 33.3) |
| Barh El-Gazel | 55.6 (50.0, 61.1) | 41.6 (35.2, 48.2) | 50.4 (44.2, 56.6) | 53.9 (47.9, 59.8) | 33.5 (28.0, 39.4) |
| Lac | 57.9 (52.8, 62.8) | 48.8 (44.0, 53.6) | 59.2 (53.2, 64.9) | 52.0 (45.1, 58.9) | 38.2 (32.3, 44.4) |
| Hadjer Lamis | 59.3 (53.6, 64.7) | 42.6 (37.0, 48.4) | 53.7 (47.4, 59.9) | 50.9 (45.0, 56.8) | 36.4 (31.4, 41.7) |
| Chari Baguirmi | 56.3 (50.9, 61.6) | 37.7 (31.7, 45.7) | 47.4 (40.1, 54.8) | 46.5 (39.6, 53.4) | 40.0 (34.6, 45.2) |
| Mayo Kebi Est | 52.4 (46.7, 58.0) | 39.5 (33.6, 45.7) | 40.3 (32.8, 48.2) | 49.6 (43.5, 55.8) | 34.5 (29.0, 40.5) |
| Mayo Kebi Ouest | 41.0 (35.2, 47.1) | 43.6 (38.7, 48.7) | 51.1 (44.6, 57.7) | 60.6 (53.5, 67.4) | 39.6 (34.1, 45.5) |
| Tandjile | 44.1 (39.8, 48.4) | 41.3 (35.6, 47.2) | 40.7 (34.4, 47.4) | 43.0 (36.5, 49.3) | 41.7 (35.7, 47.9) |
| Moyen Chari | 51.2 (45.4, 57.0) | 36.7 (31.8, 41.9) | 33.2 (27.5, 39.4) | 43.9 (37.1, 50.9) | 38.8 (33.6, 44.4) |
| Mandoul | 56.6 (52.1, 61.0) | 35.6 (30.3, 41.2) | 38.3 (32.5, 44.5) | 57.7 (50.4, 64.7) | 36.8 (30.8, 43.2) |
| Logone Occidental | 46.4 (41.9, 51.1) | 31.7 (27.0, 36.8) | 40.1 (34.5, 46.1) | 47.3 (42.3, 52.3) | 40.7 (35.0, 46.7) |
| Logone Oriental | 54.7 (50.3, 59.0) | 33.3 (27.6, 39.7) | 42.7 (37.1, 48.5) | 47.0 (41.6, 52.5) | 37.8 (32.2, 43.6) |
| N’Djamena | 37.2 (33.1, 41.4) | 34.0 (29.7, 38.6) | 41.4 (36.2, 46.7) | 34.2 (28.8, 39.9) | 28.9 (24.4, 33.9) |

CI; confidence interval
